# The Role of Somatic L1 Retrotransposition in Human Cancers

**DOI:** 10.3390/v9060131

**Published:** 2017-05-31

**Authors:** Emma C. Scott, Scott E. Devine

**Affiliations:** 1 Graduate Program in Molecular Medicine, University of Maryland, Baltimore, MD 21201, USA; escott@umaryland.edu; 2 Institute for Genome Sciences, University of Maryland School of Medicine, Baltimore, MD 21201, USA; 3 Department of Medicine, University of Maryland School of Medicine; Baltimore, MD 21201, USA; 4 Greenebaum Comprehensive Cancer Center, University of Maryland School of Medicine, Baltimore, MD 21201, USA

**Keywords:** retrotransposon, somatic retrotransposition, cancer genomics, LINE-1, L1

## Abstract

The human LINE-1 (or L1) element is a non-LTR retrotransposon that is mobilized through an RNA intermediate by an L1-encoded reverse transcriptase and other L1-encoded proteins. L1 elements remain actively mobile today and continue to mutagenize human genomes. Importantly, when new insertions disrupt gene function, they can cause diseases. Historically, L1s were thought to be active in the germline but silenced in adult somatic tissues. However, recent studies now show that L1 is active in at least some somatic tissues, including epithelial cancers. In this review, we provide an overview of these recent developments, and examine evidence that somatic L1 retrotransposition can initiate and drive tumorigenesis in humans. Recent studies have: (i) cataloged somatic L1 activity in many epithelial tumor types; (ii) identified specific full-length L1 source elements that give rise to somatic L1 insertions; and (iii) determined that L1 promoter hypomethylation likely plays an early role in the derepression of L1s in somatic tissues. A central challenge moving forward is to determine the extent to which L1 driver mutations can promote tumor initiation, evolution, and metastasis in humans.

## 1. Introduction

Transposable genetic elements constitute at least 45% of the human genome [1]. Some of these mobile elements or “jumping genes” have the ability to replicate themselves and insert new copies elsewhere in the genome [2]. The most prevalent mobile element in humans is the Long Interspersed Element 1, abbreviated LINE-1 or L1, which has expanded to over 500,000 copies in the human genome and makes up ∼17% of the human genome sequence [1]. This massive copy number expansion is the result of over 150 million years of L1 propagation that began with the incorporation of these elements into ancestral genomes sometime before the mammalian radiation [1]. L1s belong to the non-long-terminal-repeat (non-LTR) class of retrotransposons, which have certain sequence attributes and move through an RNA intermediate. Non-LTR retrotransposons themselves originated over 600 million years ago (probably from eubacterial reverse transcriptases) during the Precambrian Era and likely predate multicellular eukaryotic life [3,4]. The end result of L1 expansion over these hundreds of millions of years is a human genome that is littered with L1 copies [1]. A small number of these endogenous L1 retrotransposons remain actively mobile today and continue to mutagenize human genomes. We present here a brief review of our current understanding of how these elements influence human genomes, and ultimately, human health. A major focus is to examine somatic L1 retrotransposition as a causative agent in human cancers.

## 2. Mobilization of L1 Retrotransposons

**Figure 1:**
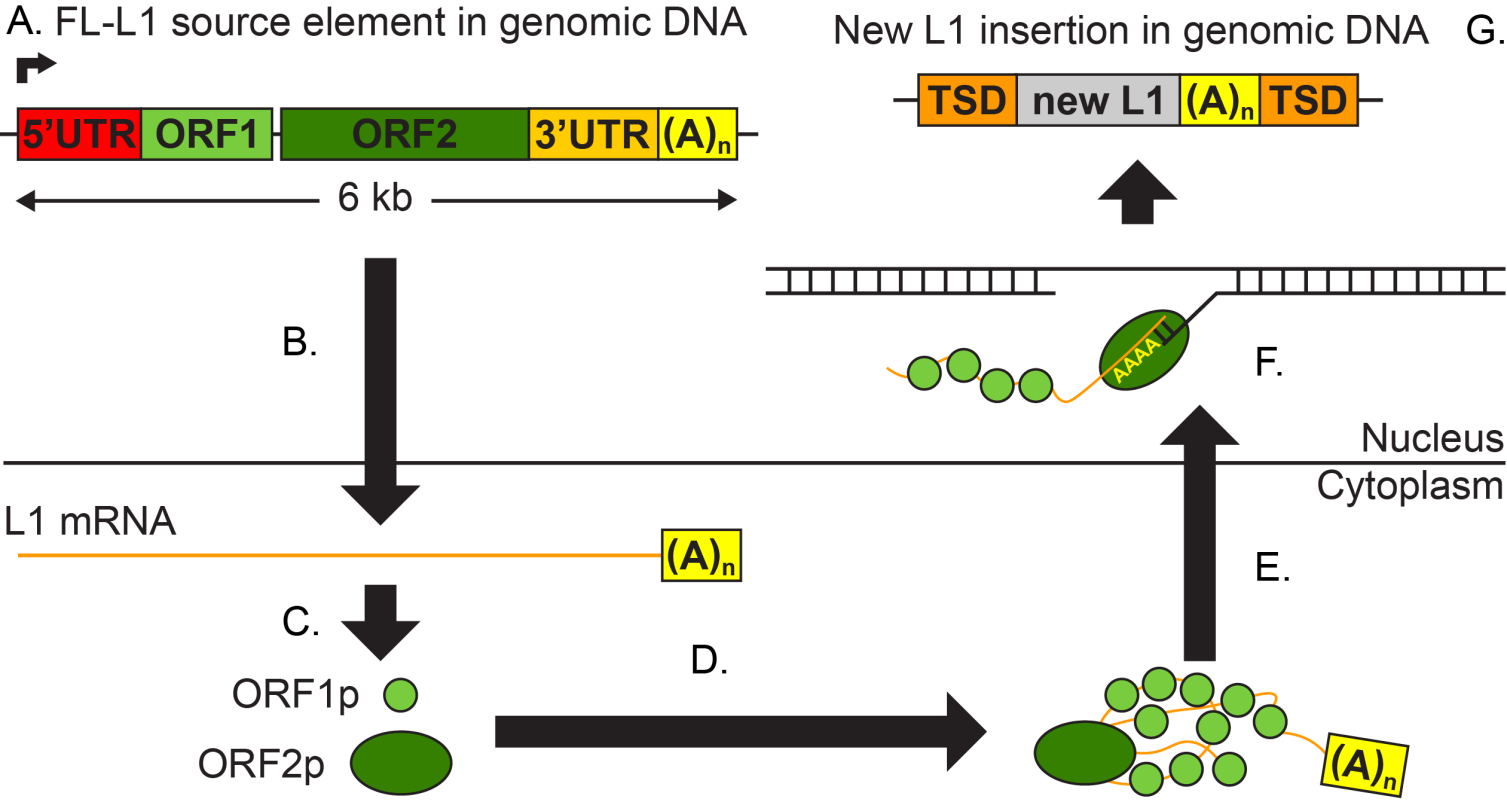
Mobilization of L1s. New L1 insertions are generated via the five step process depicted here. This process begins with a full-length (FL)-L1 source element in the genomic DNA (**A**; colored bar; L1 features are not to scale). This element is transcribed (**B**) and the resulting mRNA (orange) is exported into the cytoplasm. This mRNA is translated (**C**) into the open reading frame (ORF)1p (light green) and ORF2p (dark green) proteins, which bind the L1 mRNA to form a ribonucleoprotein complex (**D**). This complex is imported (**E**) into the nucleus. Finally, the new L1 insertion is generated by target-primed reverse transcription (**F**). The result of this mobilization process is another copy of L1 (grey) located somewhere in the genome, flanked by target site duplications (TSDs; orange) and with a poly(A) tail (yellow) (**G**). UTR; untranslated region.

In order to examine how L1s influence human genomes and disease, we must first understand how these elements are mobilized (Figure 1).

This process begins with a canonical full-length L1 (FL-L1) source element that is ∼6 kb long and consists of a promoter that is located within a 5${}^{\prime }$ untranslated region (UTR), two non-overlapping open reading frames (ORFs), a 3${}^{\prime }$ UTR, and a poly(A) tail (Figure 1A). Only a fraction of the L1 copies in the human genome have these features, as most copies are either 5${}^{\prime }$ truncated or have other deleterious mutations (see below). The first step in L1 mobilization is transcription of the FL-L1 source element from its internal promoter (Figure 1A), most likely by RNA Polymerase II [5]. This process arises only from FL-L1 source elements, since truncated L1s are missing important sequence features in the first 100 bp of the 5${}^{\prime }$ UTR that are critical for transcription initiation [6]. The resulting bicistronic mRNA is then exported from the nucleus into the cytoplasm, where it is translated to make two L1-encoded proteins, ORF1p and ORF2p (Figure 1B). ORF1p is a small RNA-binding protein [7]. ORF2p is a larger protein that encodes both endonuclease (EN) [8] and reverse transcriptase (RT) functions [9]. These proteins then bind the L1 mRNA that produced them to form a ribonucleoprotein complex [10,11] that enters the nucleus (Figure 1D,E). The ORF1p and ORF2p proteins have a strong *cis* preference for mobilizing the specific mRNA that gave rise to them, which allows for the preferential amplification of functional L1 copies [11,12,13]. Recently, a third ORF (named ORF0) was discovered in primate L1s (including humans); ORF0 is transcribed from an antisense promoter in the canonical L1 5${}^{\prime }$ UTR and can be translated into the ORF0 protein or alternative ORF0-fusion proteins that include the coding exons of neighboring genes [14]. Although ORF0 proteins can increase L1 activity (through an unknown mechanism), they are not necessary for retrotransposition [14].

The final step of L1 mobilization is known as target-primed reverse transcription (TPRT) (Figure 1F). This mechanism was first characterized in the non-LTR retrotransposon R2 from the silkworm *Bombyx mori* [15], and later was shown to accurately describe L1 integration as well [16]. Briefly, the process begins when the EN domain of ORF2p nicks the genomic DNA at its target site. The consensus recognition sequence for the EN domain is 5${}^{\prime }$-TTTT/A-3${}^{\prime }$ [8,17,18], although there is considerable flexibility in the exact site that is bound and cut by EN [19]. This cleavage exposes a 3${}^{\prime }$OH group, which is then used by the ORF2p RT domain to prime the reverse transcription of the L1 mRNA, starting from the poly(A) tail [20,21] and extending towards the 5${}^{\prime }$ end of the mRNA [15,16]. Finally, the complementary strand is synthesized and the junctions are repaired through mechanisms that are not well understood, likely involving host factors. The result of this process is a newly-inserted L1 copy, or “offspring” insertion, at a second genomic site (Figure 1G). It is important to note that the original FL-L1 source element remains intact in this process, and is capable of producing additional offspring insertions [22].

New L1 insertions have important hallmark features of retrotransposition: a poly(A) tail, flanking target site duplications (TSDs), frequent 5’ truncation, and occasional 5’ inversion [5,23,24,25,26]. Additionally, L1s can sometimes mobilize downstream sequences in a process that is known as 3’ transduction; this is thought to occur when transcription continues through the L1 polyadenylation signal and terminates after another signal in the adjacent genomic DNA [27,28,29]. The L1 retrotransposition machinery also can be hijacked by the nonautonomous retrotransposons *Alu* [30] and SVA [31,32], as well as cellular mRNAs [12]. As a consequence, the L1-mediated TPRT mechanism ultimately is responsible for the mobilization of most, if not all, recently inserted mobile elements in human genomes. Likewise, all of these mobile elements have features in common with L1 (e.g., TSDs that are similar in length and sequence composition) because they are all mobilized by the same TPRT mechanism and L1 proteins. Polymorphic copies of HERV-K elements (endogenous retroviruses) also are found in human genomes, but evidence suggests that functional HERV-K copies are either very rare or no longer exist in human populations [33,34].

The large number of individual steps that are required for L1 mobilization present numerous opportunities for human cells to regulate this mutagenic activity, and many such L1 regulators have evolved. In fact, there are mechanisms of L1 repression that act at each step of the mobilization process. For example, one of the most well-studied mechanisms of L1 repression is the methylation of CpG dinucleotides in the L1 promoter [35,36]. Promoter methylation silences L1s by repressing transcription, which is the first step of the mobilization process [36]. Beyond methylation, there are numerous other activities that inhibit L1 mobilization. These include histone modifications and chromatin remodeling (which also regulate L1 transcription), small RNA-mediated mechanisms (including piwi-interacting RNAs and small-interfering RNAs), and numerous cellular proteins that inhibit L1 mobilization post-transcriptionally in both the cytoplasm and the nucleus (reviewed recently by JL Goodier [37]). These redundant mechanisms work together to repress L1 activity.

## 3. An Historical Perspective of L1 Activity

Mobile genetic elements were first discovered in maize genomes by Barbara McClintock in 1950 [2]. This work eventually won her the Nobel Prize in Physiology or Medicine in 1983 [38], once the ubiquitous nature of transposable elements was recognized and fully appreciated. Repetitive elements were first discovered in the human genome during the course of DNA renaturation experiments in the 1960s, but these elements remained a mystery for some time [39,40]. L1 elements specifically were found in the human genome in the early 1980s [41,42,43]. From their first description by Adams et al. in 1980 it was suggested that L1s could be “essentially parasitic” DNA without a function (page 6126); this speculation was based on the lack of measurable L1 transcription in bone marrow cells [41]. This concept of parasitic or selfish DNA was not new. The exact origin of the idea is difficult to determine, but it was popularized around this time by the publication of Richard Dawkins’ book *The Selfish Gene* in 1976, and there were even ongoing reviews and debates about this notion in *Nature* in 1980 [44,45,46]. However, this classification of L1s as useless “junk DNA” was challenged as soon as it was introduced, both theoretically within the same article by Adams et al. [41] and by others as scientists began to recognize the mutagenic potential of these sequences.

During the 1980s there was a furious rush to describe the newly-discovered L1 repetitive elements. This included classifying L1s, determining their function, and beginning to understand their activity—all of which occurred in the short span of eight years. When L1s were first discovered in 1980, they were simply described as long repeated sequences of variable length that were dispersed across all human chromosomes [41]. Researchers in the field quickly determined that L1s had been discovered by multiple laboratories, and introduced standardized terminology by 1982 [42]. Long repetitive elements in general were referred to as LINEs, and the most common element was termed LINE-1 or L1. Next, researchers concluded (from many lines of evidence) that these repeats could potentially be mobile elements and speculated that they were moving through an RNA intermediate (summarized by Singer and Skowronski in 1985 [47]). In just the next year (1986), concrete evidence to confirm this hypothesis was found with the discovery that L1 ORF2 could potentially encode a protein with homology to reverse transcriptase proteins [43]. Around the same time, FL-L1 transcripts were shown to be expressed in a human teratocarcinoma cell line [48,49]. These studies provided additional support for the theory that L1s might represent mobile elements that could mutagenize the human genome, a hypothesis that would soon be confirmed.

The first evidence for ongoing mobility of L1s in human genomes came in 1988, when Kazazian et al. found two disease-causing de novo germline L1 insertions [25]. This L1 activity was discovered during a screen of hemophilia A patients for pathogenic mutations in the Factor VIII gene (*F8*) on Chromosome X. Two unrelated patients were found to have germline L1 insertions in the *F8* gene that were absent from their parents’ genomes, indicating that these L1 insertions probably occurred in gametes or during early embryogenesis [25]. The two most important findings of this study were that L1s are actively mobile in human genomes and that offspring insertions can cause disease by disrupting genes. Months after this initial discovery, a putative somatic L1 insertion was identified in a case of breast cancer [50]. This 7–8 kb insertion was located in an intron of the *MYC* proto-oncogene. However, only part of this L1 insertion was sequenced and the structure lacked the hallmarks of TPRT [51]. Thus, although this study offered the first suggestion of somatic L1 activity in humans, such activity was unlikely in this case.

The next bona fide instance of L1 retrotransposition was found in 1992, when a somatic L1 insertion was discovered by Miki et al. in a case of colorectal cancer (CRC) [52]. Similar to the L1 mutations in *F8* [25], this insertion was discovered during a screen for mutations in the *APC* tumor suppressor gene (TSG) in tumors of CRC patients. This screen was performed because *APC* plays a pivotal role in this kind of cancer: the majority of CRC tumors are initiated by mutation or loss of function of both *APC* alleles in normal colon cells, which results in the formation of a precancerous lesion that can eventually progress to carcinoma [53,54,55]. This L1 insertion was absent from adjacent normal colon tissue, suggesting that it occurred in a cancerous or precancerous colon cell sometime during the tumorigenesis process. This was remarkable because it was the first somatic L1 retrotransposition event that was documented in humans. This 750 bp insertion was flanked by TSDs and had a poly(A) tail, indicating that it was a bona fide somatic insertion produced by the TPRT mechanism. Likewise, this insertion interrupted the last coding exon of *APC*, so it was expected to disrupt normal APC function. The authors emphasized that the insertion likely initiated the tumor, but in view of our current understanding of CRC, this interpretation is somewhat unclear (see Section 5 for discussion).

For many years this was the only available evidence that L1 elements were capable of retrotransposition in somatic tissues. There was another putative example of somatic L1 activity published in 1997 [56]. However, this insertion again lacked the expected hallmarks of TPRT, and therefore, most likely was not produced by somatic retrotransposition [51,56]. Thus, genuine somatic L1 insertions appeared to be rare in humans in these early studies. In contrast, germline L1 insertions have been linked to many additional human diseases throughout the 1990s, 2000s, and to the present. In fact, germline L1 insertions have caused at least 29 cases of human disease, and have contributed to another 94 cases indirectly by mobilizing *Alu*, SVA, or other mRNAs (reviewed in [57]). In most cases, these disease-causing insertions occurred within the coding exons of known genes and disrupted gene function (although insertions in the promoters, 5${}^{\prime }$ UTR, 3${}^{\prime }$ UTR, and introns of genes also have been observed).

At this point in time scientists were undertaking important mechanistic research on L1 retrotransposons, including studies aimed at understanding how L1s are mobilized (briefly reviewed in Section 2). Much of the work during this period was done with a clever cell culture-based retrotransposition assay [58]. Specifically, Moran et al. demonstrated in 1996 that a plasmid-borne FL-L1 source element was highly active in HeLa cells, and could generate numerous offspring insertions in its host’s HeLa cell chromosomes [58]. Retrotransposition of the L1 element was dependent upon the two proteins that were encoded by the plasmid-borne L1 copy (ORF1p and ORF2p), as targeted mutations of these regions abolished L1 activity. Moreover, the new L1 insertions that were generated in HeLa chromosomes had the expected features of TPRT-mediated events. This study was remarkable because it confirmed that L1s are indeed active retrotransposons in humans [58]. However, the L1 retrotransposition assay itself is in some ways equally remarkable because it has fueled decades of productive research on L1 biology. For example, Brouha et al. [59] and Beck et al. [60] have used this assay to identify active FL-L1 source elements in human genomes. Others have used the assay to study the roles of the L1-encoded ORF0p, ORF1p, and ORF2p proteins in retrotransposition [8,14,58,61,62,63]. Conceptually similar *Alu* [30] and SVA [31,32] assays have confirmed that both of these nonautonomous elements hijack the L1 machinery for their own mobilization. The L1 assay also has been adapted for the creation of mouse models to study the timing and effects of L1 retrotransposition in the mouse [64,65,66,67,68]. Many other advances have leveraged these assays as well (reviewed in [69]).

## 4. Somatic L1 Activity in Human Genomes

As outlined above in Section 3, Miki et al. reported the earliest example of a somatic L1 insertion that might have helped to drive tumorigenesis in humans [52]. However, almost two decades passed before it became apparent that somatic L1 insertions occur frequently in human epithelial cancers. After a hiatus of 18 years, our laboratory demonstrated in 2010 that somatic L1 insertions occur frequently in human lung tumors [51]. A series of other studies subsequently revealed that somatic L1 retrotransposition is a hallmark feature of human epithelial cancers [70,71,72,73,74,75,76,77,78,79,80,81] (see Table 1). These observations have led to the suggestion that L1 might generate driver mutations in proto-oncogenes or TSGs that could fuel tumorigenesis in humans [51,52,70] (see Section 5). In the following sections, we review these studies and explore the role of somatic L1 retrotransposition in human cancers.

### 4.1. A Second Discovery of L1 Retrotransposition in Cancer

As mentioned above, we rediscovered somatic L1 retrotransposition in human tumors in a study that leveraged next-generation sequencing technologies to investigate L1 retrotransposition in human populations and cancers [51]. This study introduced the L1-Seq assay, which is a modified and updated version of L1 Display [82]. Similar to L1 Display, L1-Seq exploits sequence features of the youngest, most active L1 (L1-Ta) elements to selectively amplify the 3’ insertion junctions of these young L1 copies. In contrast to L1 Display assays, which use a gel electrophoresis step to visualize new L1 insertions, L1-Seq assays instead apply DNA sequencing technologies directly to the junction fragments to discover the chromosomal coordinates of new L1 insertions in a high-throughput manner.

After developing and optimizing the L1-Seq approach, we used it to discover new L1 insertions in 38 diverse humans, eight tumor-derived cell lines, 20 non-small cell lung tumors (with matched normal tissues), and 10 brain tumors (with matched blood leukocyte controls) [51]. This screen identified 802 novel L1 insertions, the majority of which were rare germline insertions. However, nine somatic L1 insertions also were identified in six of the 20 lung tumors, which were validated with PCR and Sanger sequencing. Somatic L1 insertions were not found in the brain tumors that were examined in this study, foreshadowing the fact that somatic L1 insertions do not occur in all tumor types (see Section 4.2). Taken together with the Miki et al. 1992 study, our study revealed that somatic L1 retrotransposition occurs in at least two types of human cancers (colon and lung), and suggested that somatic L1 retrotransposition might occur more broadly than previously appreciated [51,52].

### 4.2. Cataloguing Somatic Retrotransposition in Cancer

During the seven years that have elapsed since our 2010 study [51], many additional studies have documented somatic L1 activity in a range of human epithelial cancers. The main findings of these 14 research articles are summarized in Table 1. These papers have established many principles of somatic L1 activity, while at the same time posing important new questions.

Although these studies employed several strategies to measure somatic L1 activity, most of the methods can be grouped into two basic categories: (1) targeted resequencing assays and (2) bioinformatics tools that use whole genome sequencing (WGS) or whole exome sequencing (WES) to discover somatic L1 insertions. Targeted resequencing tools exploit specific sequence features of young L1s to selectively amplify and sequence novel L1 insertion junctions. These assays are similar to the previously described L1 Display [82] and L1-Seq [51] assays, often with further improvements in multiplexing, genome coverage, and enrichment of L1 junction fragments prior to sequencing [71,72,77,78,79,81,83,84]. With the decreased price of WGS, and the availability of such data in public repositories, several groups have developed bioinformatics tools to discover somatic L1 insertions in silico using WGS or WES data [70,74,75,80]. One advantage of this approach is that existing WGS or WES data from large consortia (like The Cancer Genome Atlas project (TCGA) [70,74,75] and the International Cancer Genome Consortium (ICGC) [75]) can be directly screened for somatic L1 insertions. As the cost of genome sequencing continues to decrease, WGS is increasingly being used to discover somatic L1 insertions in smaller laboratories as well [80]. This approach also provides the opportunity to assess other somatic variants (without any additional sequencing) to understand how L1 insertions work together with other mutagenic processes to drive tumorigenesis [75,80]. It is important to note that all of these techniques require validation to confirm that putative somatic insertions have the expected features of TPRT-mediated events (e.g., poly(A) tails and flanking TSDs).

Somatic L1 activity in cancer genomes has been found to be almost entirely confined to tumors arising from epithelial tissues [70] (see Table 1 for details). The highest levels of L1 mobilization are found in lung [51,74,75] and colorectal [70,71,74,75,78,80] cancers. Moderate L1 activity is seen in esophageal [76,79], pancreatic [77,78,81], head and neck [74,75], uterine [74], ovarian [70,74,81], gastric [78], and prostate [70,75] cancers. Lower but detectable levels of L1 activity have been seen in breast [74,75], bone [75], liver [72], kidney [74], and testicular [78] cancers. Only one TPRT-mediated somatic L1 insertion has been found in a case of brain cancer, though it is unclear whether this event occurred in the normal brain or during tumorigenesis because the normal DNA that was used for comparison was isolated from blood instead of adjacent normal brain tissue [83]. One other somatic L1 insertion was identified in a glioma tumor-derived cell line [75], though the exact timing of this insertion is again not discernible. Numerous other studies have found no such events in 60 brain tumors that were examined, leading to the consensus that somatic L1 activity in brain cancers is either absent or extremely rare [51,70,74,84]. This is somewhat ironic, given that L1s are very active in normal brain tissues (see Section 4.5). A similar lack of L1 activity has been noted in 25 examined hematologic malignancies [70,74]. These results are perhaps not surprising in light of in vitro experiments that demonstrated very low levels of engineered L1 activity in precursor cells of these cancer types (astrocytes [85] and hematopoietic stem cells [86]); these data also further support the theory that somatic L1 activity is only found in tumors of epithelial origin [70]. Within each tumor type, the number of somatic L1 insertions per tumor also can vary substantially. This is illustrated most clearly in the lung tumors that were sequenced by the ICGC, where the number of somatic L1 insertions per tumor ranged from zero to over 800, with an average of ∼63 insertions per tumor [75].

Additionally, L1s are responsible for the occasional somatic retrotransposition of other sequences in cancer genomes. The most commonly mobilized non-L1 sequences are 3${}^{\prime }$ transductions, which are produced when the sequence downstream of a FL-L1 source element is mobilized (see Section 2 and Section 4.3). In one large study, 3${}^{\prime }$ transductions occurred in 24% (655/2756) of the somatic retrotransposition events that were discovered in tumors [75]; half of these transductions were so-called “orphans” consisting only of downstream DNA without any L1 sequence [75,87,88]. These 3${}^{\prime }$ transductions can amplify exons, entire genes, and regulatory sequences, providing another mechanism by which L1s might alter the function of cancer cells [75].

Processed pseudogenes also are generated in somatic tissues via the retrotransposition of cellular mRNAs by the L1 machinery [89,90]. Such events occur at a low rate in human tumors (42 insertions in 629 tumors examined in an in-depth study of this phenomenon) [90]. Similar to L1 activity, somatic processed pseudogene formation is observed most frequently in lung and colorectal cancers, and less often in other epithelial cancers. Of note, one processed pseudogene insertion was recovered from a chondrosarcoma, which is a cartilage tumor of mesenchymal origin [90]; this finding contradicts the theory that L1 activity is restricted to epithelial tissues [70]. Infrequent somatic *Alu* insertions also have been observed in various epithelial cancers [70,71,74,75], and one somatic SVA insertion has been validated in a head and neck cancer [74]. Thus, in addition to simple L1 insertions, other non-L1 sequences are mobilized by the L1-mediated TPRT mechanism at lower frequencies in somatic tumor tissues.

### 4.3. Identification of Active Full-length (FL)-L1 Source Elements in Tumors

It is increasingly becoming possible to identify the specific FL-L1 source elements that produce somatic offspring insertions in tumors. A key development in this regard has been a much more extensive knowledge of the FL-L1 source elements that are harbored by human genomes. Based on the reference (REF) human genome sequence, Brouha et al. initially estimated that every individual has a collection of approximately 80 to 100 FL-L1 source elements that are retrotransposition-competent [59]. Later studies demonstrated that each individual also has non-reference (non-REF) FL-L1 source elements [60,80,91] (and our unpublished data). We refer to this collection of REF and non-REF FL-L1 source elements as an individual’s FL-L1 source element profile [80]. Source element profiles appear to vary considerably from one person to the next, and such differences likely produce variation in the levels of germline and somatic L1 mutagenesis that are caused by L1s [59,60,80,91] (and our unpublished data). Thus, a key goal for the future will be to understand exactly how these FL-L1 profiles vary in human populations and how this variation affects L1-mediated diseases, including cancers.

Currently, there are two available methods to determine source/offspring relationships between L1 elements. The first uses 3${}^{\prime }$ transductions to identify offspring insertions that were generated from a specific source element; this technique is very effective and accurate, but is useful only for the subset of insertions that have 3${}^{\prime }$ transductions [22,73,75]. We recently developed a second method to track source/offspring relationships using interior mutations that are frequently found in FL-L1 source elements [80]. Each source element has its own unique set of interior mutations that is inherited by offspring insertions emanating from that source element, and this provides a means to track source/offspring relationships [80].

Both of these methods have been used to study the FL-L1 elements that are active in human tumors. One large study employed the 3${}^{\prime }$ transduction method and found that a relatively small number of source elements (72) were responsible for the bulk (95%) of measurable somatic L1 activity in 290 tumors of 12 types [75]. Even more surprising, two of these source elements were responsible for over a third of the somatic activity that could be tracked with 3${}^{\prime }$ transductions [75]. Activity from one of these elements (along with many others) has been mistaken for chromosomal translocations by multiple cancer genomics groups [73,75]. This is an extremely important distinction because translocations can create novel fusion genes (which are often oncogenic) whereas L1 insertions cannot [75]. It is important to note that only ∼24% of somatic offspring can be attributed to specific source elements using 3${}^{\prime }$ transductions, and much of what we currently know about source elements in cancer is based upon such methods. Thus, additional studies will be necessary to identify all of the source elements that are somatically active in human cancers. As a step in this direction, we recently developed and used the interior mutation method to identify source elements that produced somatic L1 insertions in a case of CRC [80]. Three non-REF source elements (on Chromosomes 17, 14, and 12) produced the majority of somatic L1 insertions in this tumor, including an insertion that disrupted the *APC* TSG and initiated tumorigenesis [80].

These studies have uncovered some important features of active source elements. For instance, both REF and non-REF FL-L1 source elements can contribute to somatic L1 retrotransposition [75,80]. Source elements that are active in tumors have two intact ORFs, and most belong to the youngest L1 subfamily (Ta-1d) [80]. Some sources are “hot” L1s [75,80], which are particularly active in the cell culture-based retrotransposition assay [58] (see Section 3). The global distribution of source elements also can differ—some source elements are population-specific, while others are present in all 26 of the global populations that were examined by the 1000 Genomes Project [80]. The number of active source elements, and the amount of activity for each source element, varies considerably between tumors and tumor types. This activity can even change with the different stages of tumor evolution, with levels of L1 activity fluctuating during cancer development and progression [75,77]. In some instances, somatic FL-L1 insertions can even themselves give rise to further somatic L1 activity [75].

### 4.4. Mechanism of Reactivation of L1s in Cancer Genomes

The discovery of frequent somatic L1 activity in cancer has researchers asking why this phenomenon is occurring and why it is so variable between tumors and tissues. Promoter methylation is one of the earliest lines of defense against L1 activity, and therefore, hypomethylation is thought to be a necessary step for L1 reactivation in tumors. This hypothesis has been corroborated by many groups. These studies examined methylation at three different levels, using techniques that vary in scope. First, the Illumina Infinium platform assesses global genomic methylation within CpG islands [51]. Second, average L1 promoter methylation across all FL-L1 copies can be measured using general bisulfite sequencing PCR (with internal L1 primers that amplify most FL-L1 promoters [71,72]). Finally, methylation of specific FL-L1 source elements can be measured with targeted bisulfite sequencing PCR by amplifying the 5${}^{\prime }$ junctions of these elements (using a primer in the upstream genomic DNA in combination with an internal L1 primer) [75,80].

Using the first method (the Illumina Infinium platform), we identified a hypomethylation signature at 59 genomic CpG sites that was associated with L1-permissive lung cancers [51]. Soon thereafter, Solyom et al. identified four CpG sites in L1 promoters that were hypomethylated in CRC tumors compared to normal colon tissue [71]. Hypomethylation of the entire CpG island was later observed in the L1 promoters of liver tumors compared to adjacent normal tissue [72]. Moreover, there was a strong positive correlation between the level of hypomethylation at these promoters and the number of somatic L1 insertions that were produced in all three tumors that were examined [72]. These last two studies used methods that measure the average methylation levels at many L1 promoters throughout the genome (i.e., the second, general method listed above). In contrast, two recent studies have inspected the promoters of specific FL-L1 source elements using the third (targeted) method outlined above. These studies found that the source elements (i) were hypomethylated in the tumors in which they caused somatic L1 insertions; (ii) were usually methylated in tumors that lacked activity from the source element; and (iii) were often methylated in normal tissue [75,80]. The functional consequences of L1 promoter hypomethylation also have been demonstrated—a source element that produced a somatic insertion early in tumorigenesis was hypomethylated and transcribed in both normal and tumor colon tissue [80]. Collectively, these results suggest that methylation represses L1 elements in normal somatic tissues, but is either absent or removed from elements that have become reanimated. Other host factors and mechanisms likely contribute to this process as well (see Section 2). Additional work is needed to better understand how these elements are silenced and derepressed in somatic human tissues, and how these processes impact tumorigenesis.

### 4.5. L1 Retrotransposition Contributes to Genomic Diversity in the Adult Brain

In addition to cancer genomes, somatic retrotransposition also occurs in neuronal cells [66,85] and normal brain tissues [66,85,92]. Using an array capture and sequencing-based technology to discover new L1 insertions, the Faulkner group documented high levels of L1 activity in the human brain (850 putative somatic L1 insertions in three individuals) [92]. Somatic retrotransposition was confirmed in the brain independently by the Walsh lab using single cell sequencing technology, although at much lower levels [93]. The Faulkner lab subsequently carried out their own single cell analyses in human neuronal brain tissues, and verified that somatic L1 retrotransposition indeed occurs at high frequencies in such tissues [94] (in agreement with their earlier 2011 study [92]). Although there is some disagreement about the absolute level of somatic retrotransposition that occurs in the human brain, these studies seem to agree that such mobilization occurs. While this is not directly related to cancer, it is important to consider that somatic mobilization is not limited to the germline and tumors, but also occurs in the brain and may occur in other normal somatic tissues as well (see Section 6.2). It is of interest to note that the first transposable element that was discovered by Barbara McClintock is mobilized exclusively in the somatic cells of maize [2].

## 5. L1 as a Driver of Tumorigenesis

Perhaps the most important discussion ongoing in the human retrotransposon field is the exact timing of L1 activity during tumor development and progression. This boils down to one central question: are somatic L1 insertions drivers of tumorigenesis or mere passengers along for the ride? Nearly every published paper in the field addresses this question, including the first documented case of somatic L1 activity from 1992 [52]. This somatic L1 insertion disrupted a coding exon of the *APC* TSG and likely was a driver of tumorigenesis [52] (Table 2). However, in view of our current understanding of CRC, the precise role of this insertion in tumorigenesis remains unclear. This is because the second *APC* allele was not examined in that study and the authors did not rule out a possible role for faulty DNA repair as an initiator of tumorigenesis [52] (see [80] for details). Thus, the precise stage of tumorigenesis that was affected by this L1 driver mutation (i.e., tumor initiation vs. a later stage) remains unclear. There have been additional findings supporting the theory that somatic L1 insertions participate in tumorigenesis. For example, a somatic L1 mutation has been identified in a uterine tumor in the known cancer gene *PTEN* (Table 2). However, a relatively small number of clear L1 driver mutations have been discovered to date (Table 2; see Section 6.1 for further discussion of this point).

Studies published in 2012 [70] and 2014 [74] confirmed that intronic L1 insertions in human tumors usually result in decreased expression of the mutated genes, and thus, could theoretically contribute to tumorigenesis by decreasing the expression of TSGs. However, the frequency of this occurrence is under debate: two additional studies had the contradictory finding that the vast majority of intronic somatic L1 insertions had no effect on gene expression in liver, lung, and colon tumors [72,75]. Thus, although L1 insertions can change gene expression in tumors at least occasionally, more work is needed to reach a consensus on how frequently this occurs. Interestingly, the unique type of mutation that is caused by L1 mobilization (i.e., structural variation) also can have unexpected effects on gene expression. For example, Shukla et al. [72] identified a somatic L1 insertion within an intron of the *ST18* gene that disrupted a *cis*-regulatory repressor element, which in turn led to increased expression of the *ST18* gene (Table 2). This group concluded that *ST18* is likely a proto-oncogene that has a role in driving liver cancer in this case [72]. It has since been demonstrated that *ST18* is important for the development and persistence of liver tumors by facilitating interactions with tumor associated macrophages [95]. Therefore, the somatic L1 insertion in *ST18* likely was integral for the formation and maintenance of the tumor in which it was discovered [72]. Both somatically-acquired and germline L1 elements in the genome also can have large effects on the transcriptome through a number of diverse mechanisms that are independent of L1 activity [96] (recently reviewed in [97]). It can be imagined that these mechanisms could impact the expression of proto-oncogenes and TSGs, adding another level of complexity of the potential roles of retrotransposons in cancer cells [72,75].

One major unresolved question is: how often do L1 driver mutations initiate tumorigenesis in normal cells? Insertions that occur after tumor growth is well underway would not appear to participate in tumorigenesis per se, but instead might play a role in tumor evolution or metastasis. Doucet-O’Hare et al. examined the evolution of esophageal cancer from a precancerous condition and demonstrated that L1s can be active very early during the tumorigenesis process, and even found somatic L1 insertions in precancerous lesions that never progressed to cancer (over a 15 year period) [79]. In a similar study, Ewing et al. also found somatic L1 insertions in precancerous lesions (adenomas) of the colon and in normal colon tissue [78]. These papers together strongly suggest that tumor-initiating L1 insertions could occur in a normal cell and then become amplified into a tumor through selection.

We recently demonstrated that L1 indeed can initiate tumorigenesis in normal colon cells [80]. *APC* is a gatekeeper TSG that is frequently mutated in patients with CRC—in fact, both copies of *APC* must be mutated to initiate most cases of CRC [53,54,55]. We discovered a somatic L1 insertion that disrupted the last coding exon of this gene [80], only 388 bp upstream of the somatic L1 insertion that was discovered by Miki et al. in 1992 [52] (Table 2). Importantly, we also established that the second *APC* allele was inactivated by a point mutation, and that this tumor had stable microsatellites, indicating that faulty DNA repair did not initiate tumorigenesis in this case [80]. Instead, we showed that the tumor developed in concordance with the well-established two-hit genetic model for CRC wherein both *APC* gatekeeper alleles are mutated in a normal cell [53,54,55]. L1 disrupted one of the two *APC* alleles and a stop codon disrupted the second allele. Thus, somatic L1 mutations can initiate tumorigenesis in normal colon cells.

## 6. Closing Remarks and Future Directions

This review has summarized the current literature documenting somatic L1 retrotransposition in human cancers. The wave of papers that has been published on this topic over the last seven years has provided a survey of which tumor types are permissive for somatic L1 activity; the field also has begun to address the important question of why this phenomenon is occurring and the effects it has on cancer development and progression. However, in the course of discovering important characteristics of somatic L1 activity, these papers also have raised several new questions that need to be addressed. To close this review, we summarize below some unsettled questions and future directions.

### 6.1. Why Don’t We See L1-Initiated Cancers More Frequently?

As discussed in Section 5, there have only been a few reported instances of probable L1-initiated tumors (Table 2). Though somatically-mobilized L1s can indeed initiate and drive tumorigenesis [80], there have been a notably small number of such cases discovered, especially considering the total number of tumors that have been examined for somatic L1 activity across all the studies in Table 1. This raises the question: why don’t we see this phenomenon more frequently? Although one possibility is that L1s initiate tumorigenesis in somatic cells only rarely, as suggested by multiple groups [71,75,77], there also are a few reasons that we may be underestimating the frequency L1-mediated cancers.

First and foremost, the genetic pathways for tumor development have not been thoroughly defined for most tumor types. Thus, we might be finding somatic L1 insertions in proto-oncogenes and TSGs that have not yet been discovered, and as a consequence, we cannot yet link these insertions to tumor development. In this regard, somatic L1 insertions may define a novel set of proto-oncogenes and TSGs that can only drive tumorigenesis when mutated by L1. In support of this idea, recurring L1 mutations have been identified in several novel genes that were not previously linked to tumorigenesis [70,71,74,75,76,78]. This phenomenon is reminiscent of studies in mice where tumors were induced by the Sleeping Beauty transposon [98]. Even when an L1 insertion occurs within a known proto-oncogene or TSG, it can be difficult to link the insertion unambiguously to the tumor in which it was discovered. For example, in some cases a known proto-oncogene or TSG might not have been linked to a specific tumor type (e.g., the role of *ST18* in liver cancer development was determined a few years after the L1 insertion in this gene was reported [72,95]). Even if the gene has been clearly implicated in the tumor type previously, it can be difficult to interpret the impact of some L1 insertions without extensive experimentation (e.g., it is difficult to predict the functional consequences of intronic insertions, such as the one that was discovered in *BRCA1* in a case of ovarian cancer [81]).

Another confounding factor is that the temporal order in which gene mutations occur during tumor initiation and evolution is unclear in most tumor types (recently reviewed in [99]). In many cases, the discovery of tumor progression pathways is hindered by the extraordinary mutational heterogeneity that is found within most cancer types [100]. As a result, there are only a few tumor types (e.g., CRC) in which we can currently determine whether tumors are actually initiated by L1 insertions in known driver genes. Thus, although projects such as TCGA and ICGC have begun to explore the mutational landscapes of many human cancers, much more work is needed to fully understand how L1 mutagenesis contributes to tumor formation.

### 6.2. To What Extent Are L1s Active in Normal Noncancerous Cells and Tissues?

Although L1 clearly is quite active in the normal somatic tissues of the brain and epithelial cancers, we are just beginning to explore the extent of somatic activity in other normal tissues. Several lines of evidence suggest that L1 can evade somatic repression in at least some other normal tissues. In one study, 21 putative somatic L1 insertions were identified in normal liver cells using a targeted sequencing assay [72]. In another study, two somatic L1 insertions were discovered in normal stomach cells [78]. Yet another study found nine putative somatic L1 insertions in the normal esophagus [79]. In all three of these studies, independent validation of somatic L1 activity in the normal tissue was either very limited or not possible. A confounding factor was that germline insertions could appear to be new somatic insertions in adjacent normal tissue if the tumor underwent chromosomal loss over the site. Regardless, these studies are likely to have reported true somatic insertions, since it can be reasonably expected that germline insertions would be detected by PCR in the tumor-infiltrating noncancerous cells that comprise the tumor stroma. Thorough validation might have been possible if a second normal tissue were available in these studies (however, this was not the case). In contrast, two additional somatic L1 insertions have been identified in normal tissues that were fully validated by PCR: the first in normal colon and absent from liver cells [78]; the second in normal esophagus (and a precancerous lesion) and absent from blood cells [79]. Finally, in our CRC study we determined that a tumor-forming L1 insertion in *APC* occurred at the earliest stages of tumorigenesis (most likely in a normal colon cell), providing further evidence that normal colon tissues can support somatic L1 activity [80]. Thus, there is a lot of evidence in the literature (albeit sometimes preliminary in nature) to suggest that normal adult tissues may broadly support somatic L1 retrotransposition.

On the basis of this limited evidence, is it possible that most (if not all) normal epithelial tissues support L1 activity? If so, this might have been largely missed for the same reason that it was initially overlooked in the brain: that each cell in a given tissue generates a unique collection of somatic L1 retrotransposition events that cannot be detected in bulk tissue. The solution to this problem is to adapt L1 discovery methods to single cell sequencing technologies. This approach has been pioneered in brain tissues by the Walsh [93] and Faulkner [94] labs, and should be adaptable to other normal tissues as well. Through whole genome amplification, this technique is able to both sequence the genome of a single cell and also provide material for validation [93,94]. Although this approach is still in its infancy, it likely will be useful for finding the somatic L1 insertions that the literature suggests are mutagenizing the genomes of normal cells throughout the human body. Clearly, more work will be necessary to determine whether this is the case.

### 6.3. How Does Inter-Individual Genomic Variation Affect Somatic L1 Activity?

Finally, we need to address how differences in FL-L1 source element profiles influence tumorigenesis. As discussed in Section 4.3, each individual inherits a different collection of FL-L1 source elements. The content of these profiles could have considerable effects on the risk of an individual developing L1-mediated diseases, including cancers. In this regard, many questions remain unanswered: How many hot FL-L1 source elements are present in each human’s genome and how does this vary from one human to the next? How many elements can evade somatic repression and initiate human cancers in normal somatic tissues, and in which tissues does this occur? This is a particularly important question because it is quite possible that most of the somatic L1 insertions that have been discovered in tumors thus far were produced by source elements that only became derepressed after tumorigenesis was underway. If this were the case, it might help to explain why the community has identified only a handful of clearly recognizable L1 driver mutations in human cancers: most insertions were generated too late to initiate or drive tumorigenesis. However, several lines of evidence indicate that at least some FL-L1 source elements can evade somatic repression in normal tissues, and generate driver mutations sufficiently early to initiate tumorigenesis. We need to explore this class of events more carefully and determine how often source elements can generate tumor-initiating mutations in normal cells. Events that occur later in tumorigenesis also need to be explored further for roles in tumor evolution and metastasis.

We also need to gain a better understanding of how FL-L1 source element profiles vary in human populations. Although some FL-L1s are ubiquitously found in most or all human genomes, many others are found only in a subset of individuals and are inherited in a population-specific manner [80]. As a result, ancestry could play an important role in determining an individual’s mutagenic burden from germline and somatic L1 activity. For example, in our CRC study, a population-specific FL-L1 source element on Chromosome 17 of the patient’s genome initiated tumorigenesis [80]. Since this element is restricted to populations that are associated with the African diaspora, the cancer risk that is associated with this element also would be restricted to such populations. At the present time, very little is known about how source element profiles vary across diverse human demographies, and how these differences affect tumorigenesis.

### 6.4. Conclusions

We have presented a brief overview of research examining somatic L1 retrotransposition in human genomes, focusing on landmark studies outlining the activity of these mobile elements in human cancers. Researchers in this field have characterized many aspects of somatic L1 activity in the short span of only seven years. However, several major questions remain unresolved. One unsettled question is: How often does somatic L1 retrotransposition initiate and drive tumorigenesis in humans? Despite the fact that thousands of somatic L1 insertions have been recovered from many tumor types, only a handful of clearly-recognizable driver mutations have been discovered. We also have a very incomplete understanding of L1 activity in normal somatic tissues. If L1s are highly active in most adult somatic tissues, we clearly need to gain a better understanding of the consequences of this activity. Likewise, in our current era of human genomics and precision medicine, it is increasingly important to define inter-individual variation and determine how variation in FL-L1 source element profiles may impact human health and disease.

## Figures and Tables

**Table 1:** Studies of somatic L1 activity in cancer genomes. Column three gives the total number of somatic L1 insertions discovered in the total number of tumors assayed; these estimates take validation rates into account when applicable.

Reference	Tumor Type	Insertions (Tumors)	Important Findings
Miki et al. 1992 [52]	Colorectal	1 (1)	First genuine somatic L1 activity; L1 insertion in *APC* might have initiated colorectal cancer (CRC), but somewhat unclear
Iskow et al. 2010 [51]	Lung, brain	8, 0 (20, 10)	Introduced high-throughput L1-Seq assay; Established that somatic L1 activity occurs frequently in lung tumors, but not in brain tumors; Suggested that L1s might drive tumorigenesis; Found a hypomethylation signature that distinguishes L1-permissive lung tumors
Lee et al. 2012 [70]	Colorectal, prostate, ovarian, brain, blood	178 (43)	Somatic L1 activity only in epithelial tumors, absent from brain and blood; Genes with somatic L1 insertions typically had decreased expression; Compared features of somatic and germline L1s
Solyom et al. 2012 [71]	Colorectal	72 (16)	Positive correlation between patient age and number of somatic L1s; Most L1 insertions occurred after tumor initiation
Shukla et al. 2013 [72]	Liver	12 (19)	Intronic somatic L1 insertion into a regulatory element increased expression of candidate liver oncogene *ST18*; Suggested that L1s might be somatically active in normal liver cells
Pitkänen et al. 2014 [73]	Colorectal	83 (92)	All L1 insertions originated from one source element on Chromosome 22, in *TTC28*; These L1 insertions were previously mischaracterized as translocations
Helman et al. 2014 [74]	11 types	695 (976)	Somatic L1 insertion in an exon of the *PTEN* tumor suppressor gene (TSG); Lung, colorectal, head and neck, and uterine cancers had highest L1 mobilization levels
Tubio et al. 2014 [75]	12 types	2711 (290)	3${}^{\prime }$ transductions make up 24% of somatic L1 activity; A small number of source elements gave rise to most L1 insertions with transductions; Active sources had promoter hypomethylation; Activity of sources fluctuates over the course of tumor evolution
Paterson et al. 2015 [76]	Esophageal	5108 (43)	The majority of L1s were discovered by searching for somatic poly(A) insertions, so some probably represent L1-mediated transposition of non-L1 sequence; Identified active source elements using 3${}^{\prime }$ transductions
Rodić et al. 2015 [77]	Pancreatic	409 (20)	Inverse correlation between survival and both the number of somatic L1 insertions and ORF1p protein expression; Retrotransposition occurs throughout tumor development, but is discontinuous
Ewing et al. 2015 [78]	Colorectal, pancreatic, gastric, testicular	104 (18)	Frequent somatic L1 insertions in precancerous adenomas; Most somatic L1 insertions were clonal; Validated one somatic non-germline L1 insertion in normal colon; Suggested that L1 insertions are occurring in normal colon or very early in tumorigenesis
Doucet-O’Hare et al. 2015 [79]	Esophageal	118 (20)	Found somatic L1 insertions in patients with Barrett’s Esophagus (a cancer-predisposing condition) and esophageal cancer; L1 activity seen in patients that did not develop cancer; Suggested that somatic L1 activity could occur in normal or metaplastic cells
Scott et al. 2016 [80]	Colorectal	27 (1)	An L1-initiated CRC caused by L1 mutagenesis of *APC* TSG; Tumor initiated by activity of a hot, population-specific FL-L1 source element, which was hypomethylated and expressed in normal colon tissue; Demonstrated that L1s can evade somatic repression and initiate tumorigenesis
Achanta et al. 2016 [83]	Brain	1 (10)	Found one somatic L1 insertion in a secondary glioblastoma; Cannot rule out that this occurred in normal brain because compared to DNA from blood
Carreira et al. 2016 [84]	Brain	0 (14)	Could only validate one TPRT-independent somatic L1 insertion and one likely *Alu-Alu* recombination event; Conclude that L1 retrotransposition does not occur in primary glioblastoma or glioma
Tang et al. 2017 [81]	Ovarian; pancreatic	35, 205 (8, 13)	Found one somatic L1 insertion in *BRCA1* TSG intron, in an ovarian cancer; Some pancreatic L1 insertions (76) were discovered in an earlier analysis of this same sequencing data [77] and used for methodological validation here

**Table 2:** Likely driver mutations caused by somatic L1 retrotransposon insertions in known proto-oncogenes and TSGs.

Gene	Location of Insertion	Tumor Type	Reference
*APC*	16th exon (coding)	Colorectal	Miki et al. 1992 [52]
*APC*	16th exon (coding)	Colorectal	Scott et al. 2016 [80]
*PTEN*	6th exon (coding)	Uterine	Helman et al. 2014 [74]
*ST18*	Intron (repressor)	Liver	Shukla et al. 2013 [72]

## Abbreviations

The following abbreviations are used in this manuscript:

L1	Long Interspersed Element 1
LTR	long-terminal-repeat
FL-L1	full-length L1
UTR	untranslated region
ORF	open reading frame
EN	endonuclease
RT	reverse transcriptase
TSD	target site duplication
TPRT	target-primed reverse transcription
CRC	colorectal cancer
TSG	tumor suppressor gene
WGS	whole genome sequencing
WES	whole exome sequencing
TCGA	The Cancer Genome Atlas
ICGC	International Cancer Genome Consortium
REF	reference
